# Comprehensive Transcriptome Analysis of Sex-Biased Expressed Genes Reveals Discrete Biological and Physiological Features of Male and Female *Schistosoma japonicum*

**DOI:** 10.1371/journal.pntd.0004684

**Published:** 2016-04-29

**Authors:** Pengfei Cai, Shuai Liu, Xianyu Piao, Nan Hou, Geoffrey N. Gobert, Donald P. McManus, Qijun Chen

**Affiliations:** 1 MOH Key Laboratory of Systems Biology of Pathogens, Institute of Pathogen Biology, Chinese Academy of Medical Sciences & Peking Union Medical College, Beijing, P.R. China; 2 Molecular Parasitology Laboratory, QIMR Berghofer Medical Research Institute, Queensland, Australia; 3 Key Laboratory of Zoonosis, Shenyang Agriculture University, Shenyang, P.R. China; University of Cambridge, UNITED KINGDOM

## Abstract

Schistosomiasis is a chronic and debilitating disease caused by blood flukes (digenetic trematodes) of the genus *Schistosoma*. Schistosomes are sexually dimorphic and exhibit dramatic morphological changes during a complex lifecycle which requires subtle gene regulatory mechanisms to fulfil these complex biological processes. In the current study, a 41,982 features custom DNA microarray, which represents the most comprehensive probe coverage for any schistosome transcriptome study, was designed based on public domain and local databases to explore differential gene expression in *S*. *japonicum*. We found that approximately 1/10 of the total annotated genes in the *S*. *japonicum* genome are differentially expressed between adult males and females. In general, genes associated with the cytoskeleton, and motor and neuronal activities were readily expressed in male adult worms, whereas genes involved in amino acid metabolism, nucleotide biosynthesis, gluconeogenesis, glycosylation, cell cycle processes, DNA synthesis and genome fidelity and stability were enriched in females. Further, miRNAs target sites within these gene sets were predicted, which provides a scenario whereby the miRNAs potentially regulate these sex-biased expressed genes. The study significantly expands the expressional and regulatory characteristics of gender-biased expressed genes in schistosomes with high accuracy. The data provide a better appreciation of the biological and physiological features of male and female schistosome parasites, which may lead to novel vaccine targets and the development of new therapeutic interventions.

## Author Summary

Schistosomiasis is a persistent but neglected parasitic disease, afflicting more than 200 million people worldwide. Complex gene regulatory mechanisms are equipped by its causative reagents, parasites of the genus *Schistosoma*. Dissecting these mechanisms thus will be beneficial for better control of the disease. DNA microarrays are flexible tools for profiling gene expression. Here, a custom printed microarray with a comprehensive coverage of the *Schistosoma japonicum* transcriptome, was utilised to decipher gender-associated genes of that species. A total of 685 and 430 mRNA transcripts were shown to be highly expressed in adult males and females, respectively. Genes enriched in the male adults were associated with cytoskeleton, motor and neuronal activities, whereas genes expressed more highly in female parasites were involved in amino acid metabolism, nucleotide biosynthesis, gluconeogenesis, glycosylation, cell cycle processes, DNA synthesis and genome fidelity and stability. A general scenario on how miRNAs potentially modulate these gender-associated genes is provided. The results here further highlight the transcriptomic differences between male and female parasites and provide a stepping-stone for identifying new vaccine and drug targets.

## Introduction

Schistosomiasis, caused by infection with blood flukes (digenetic trematodes) of the genus *Schistosoma*, remains one of the most serious parasitic diseases worldwide, afflicting more than 200 million people, with close to 800 million at risk [1,2]. This debilitating disease causes an annual number of disability-adjusted life years (DALYs) lost of up to 3.3 million in 2010, ranking it as third in the list of global neglected diseases [3]. Three main species, *Schistosoma mansoni*, *S*. *haematobium* and *S*. *japonicum* are of clinical relevance. Currently, no practical anti-schistosome vaccine is available and mass chemotherapy with a single effective drug, praziquantel, combined with morbidity management, are the primary strategies adopted for the treatment and control of schistosomiasis [4,5].

Schistosomes have a complex lifecycle involving an aquatic snail as an intermediate host and a mammalian definitive host [2]. In contrast to other trematode species, these parasites are unique in that they exhibit sexual dimorphism, and they thus represent a valuable model for invertebrate conjugal biology research. The availability of schistosome transcriptome [6,7] and genome sequences [8–10] for the three major schistosome species, provides a wealth of resources to allow the dissection of gene profiles during development and between the sexes. In this respect, a variety of high-throughput techniques have been widely employed in the study of schistosomes, including the use of microarrays [11–16], serial analysis of gene expression (SAGE) [17–19], digital gene expression (DGE) [20], and RNAseq [21,22] with each method presenting distinct advantages and disadvantages. These pioneering studies revealed expression patterns and features of developmental-, gender-dependent, tissue-specific, strain-specific and host-associated gene expression within schistosome parasites [11,13,23–25], which have contributed substantially to our understanding of their biology. However, SAGE and DGE suffer from quantifying low abundance mRNA transcripts [20], both of which may omit genes responsible for vital functions present within tissue-specific expression. This is a particular concern given that schistosomes are multi-cellular organisms. The interpretation of DNA microarray results depends on the quality of genetic information contained within the DNA sequences used for probe design. The first generation DNA microarrays used for schistosome studies were designed based on EST transcripts and the data obtained from these chips only provide a compromise interpretation due to the poor annotation of these sequences [11–13]. To provide more comprehensive gene profiles during development or between the sexes of schistosomes, a second generation DNA microarray with a well-curated design of probes based on transcriptomic and genomic sequences is required.

MicroRNAs (miRNAs) are small non-coding RNA molecules, which exert important gene regulatory functions at the post transcriptional level. The identification of schistosome miRNAs has been carried out over the past five years using techniques including cloning methods to deep-sequencing. Comprehensive miRNA expression profiles within several discrete developmental stages of schistosomes, as well as between different sexes have been presented with high accuracy and coverage [26–31]. In humans, it has been estimated that miRNAs may regulate the expression of ~60% of the total coding mRNA transcripts [32]. Plausible regulatory roles in the development and sexual maturation of schistosomes have been suggested for several miRNAs [26,27]. However, comprehensive functional annotation for individual schistosome miRNAs is still unavailable. *In silico* prediction represents a high-through approach to achieve this objective, but different prediction tools with different scoring criteria, have led to differing outputs with variable false positive and false negative rates. Recently, a novel experimental approach, using high-throughput sequencing of RNA isolated by cross-linking and immunoprecipitation (HITS-CLIP), has been used to identify miRNA targets in adult *S*. *japonicum* worms, although the results from this study were inconclusive [33].

We have constructed a second generation DNA microarray for transcriptomic study of *S*. *japonicum* based on *S*. *japonicum* and *S*. *mansoni* genomic and transcriptomic sequences with multiple probes designed against each target sequence (both forward and reverse) [34–36]. By employing this powerful microarray printed with the most comprehensive coverage of probes, we focused on the identification of sex-biased expressed genes and predicted potential miRNA targets against these genes. The study presents a global view of the expressional and regulatory features of gender-associated genes in *S*. *japonicum*, and provides novel insights on schistosome conjugal biology.

## Materials and Methods

### Ethical statement

All procedures performed on animals within this study were conducted following animal husbandry guidelines of the Chinese Academy of Medical Sciences and with permission from the Experimental Animal Committee (Institute of Pathogen Biology, CAMS) with Ethical Clearance Number IPB-2011-6.

### Parasite materials

*S*. *japonicum*-infected *Oncomelania hupensis* were provided by Hunan Institute of Parasitic Diseases, Yueyang, China. Cercariae were shed from these snails under light stimulation and used to percutaneously infect female New Zealand rabbits. Mixed adult worms were also obtained from infected rabbits by perfusion at 6 weeks post-infection (p.i.). Male and female worms were separated manually with the aid of stereomicroscope [27]. All parasite samples were soaked in RNAlater (Ambion, CA, USA), and stored at -80°C until total RNA extraction.

### Total RNA isolation

Total RNAs were isolated from male and female *S*. *japonicum* using RNeasy Mini kits (QIAGEN, GmbH, Hilden, Germany) according to the manufacturer's instructions. Potential contaminating genomic DNA was removed from RNA samples using Turbo DNA-free kit (Ambion, CA, USA). The quantity and quality of the RNA samples were assessed by a NanoDropND-1000 spectrophotometer (NanoDrop Technologies, Wilmington, DE) and denaturing agarose gel electrophoresis.

### Microarray construction and hybridization and subsequent data analysis

A schistosome genome-wide microarray was employed for analysing the gene expression profiles of male and female *S*. *japonicum* with three biological replicates. The details regarding the design and construction of the microarray, the hybridization method, and feature extraction have been reported previously [34–38]. Briefly, a total of 20,194 *S*. *japonicum* target sequences collected for creating an array. For each target sequences, 3 or 4 pairs of 60-mer complementary oligonucleotide probes (forward and reverse probes) were designed. Probes with random sequences were printed as negative controls (background signal) and eight spike-RNA probes from the intergenic sequence of yeast were used as hybridization controls. Microarrays were printed in a 12×135 K feature format (Roche NimbleGen) with a total of 145,000 probes representing 41,982 features. cDNA was labelled with a fluorescent dye (Cy3-dCTP) using a cRNA Amplification and Labelling Kit (CapitalBio, Beijing, China) [39]. Hybridization was performed using three biological replicates for all samples (CapitalBio, Beijing, China). Procedures of array hybridization, washing, scanning, and data acquisition were carried out according to the NimbleGen Arrays User’s Guide. The arrays were scanned using a MS200 scanner (NimbleGen Systems) at 2-μm resolution, and NimbleScan software (NimbleGen) was used to extract fluorescent intensity raw data from the scanned images. Normalized gene expression data were generated using the Robust Multichip Average (RMA) algorithm [40,41]. Outlier probes were identified and their contribution was reduced at the reported gene expression level, a process which has been shown to improve the sensitivity and reproducibility of microarray results [41]. Then, the expression value of a gene is a weighted average of all forward or reverse probe sets when both background correction and quantile normalization are performed. Raw data and the normalized data have been deposited at the public domain Gene Expression Omnibus under the accession number for the platform GPL18617, and series GSE57143.

### Bioinformatics analysis on sex-differentially expressed genes

Potential gender-biased expressed genes of *S*. *japonicum* were initially retrieved from the NCBI database (http://www.ncbi.nlm.nih.gov/sites/batchentrez) based on fold-changes (FC) of the mean of the weighted intensity value of forward or reverse probe set between genders (FC ≥ 2, three biological replicates). Genes were further considered differentially expressed by FC values from both forward and reverse probe sets ≥ 2 between genders (*p*<0.05, Student’s *t*-test [35,42], without *p*-value adjustment for multiple testing); only those genes with a mean of signal intensity >100 at least in one gender were included for further investigation. Heat maps were created based on the signal intensities of forward gene or EST sequences using HemI 1.0 software [43]. Gene sets were then functionally annotated using Blast2GO [44]. The gene collection was re-annotated using the BLAST program based on the annotation of their homologous sequences from *S*. *mansoni*, *S*. *haematobium*, *Clonorchis sinensis* and *Echinococcus granulosus*, deposited in NCBI database. For hypothetical proteins, conserved protein domains were further searched against the NCBI CDD database (v3.14) [45] for possible improved annotation.

### Quantitative real-time PCR

A total of 50 gender-associated and 10 non-gender-associated genes were selected for validation using qRT-PCR as described previously [34]. One microgram male or female total RNA were reverse transcribed into first-strand cDNA using a SuperScript III Reverse Transcriptase Kit (Invitrogen) with oligo (dT) 15 primer according to the manufacturer's instructions. The cDNA products were diluted 20-fold with nuclease-free water before undertaking the qPCR. Each 25 μl PCR reaction contained 12.5 μl of 2×Brilliant II SYBR Green QPCR Master Mix (Agilent, USA), 1 μl cDNA, 1 μl of the forward and reverse primer pair (S1 Table), and 10.5 μl of sterile water. PCR cycling conditions were as follows: 95°C for 10 min, followed by 40 cycles of 30 s denaturation at 95°C and 1 min annealing and extension at 60°C. A dissociation step (95°C for 15 s, 60°C for 1 min, 95°C for 15 s, and 60°C for 15 s) was performed to confirm the amplification specificity for each gene. 26S proteasome non-ATPase regulatory subunit 4 (PSMD4), a reliable reference gene for transcriptomic analysis of *S*. *japonicum* [34,46], was employed as a control gene in the assays. The PCR primers were designed using Primer Express 3.0 software (Applied Biosystems, Foster City, USA). PCR reactions were performed in technical triplicates on the 7300 Real-Time PCR system (Applied Biosystems). The relative expression level of each gene was analysed using SDS 1.4 software (Applied Biosystems). Melt curves for the genes tested are shown in S1 Fig Correlations between the microarray and qPCR results for 50 gender-associated genes were checked with the Spearman’s correlation coefficient.

### miRNA target prediction

The miRNA target sites were predicted using PITA [47], and RNAhybrid [48]. Gene sequences were downloaded from the NCBI website; mature miRNAs were downloaded from the miRBase (release 19.0) (TPM (transcripts per million) >10 in adult male and female worm libraries [26]). Target sites were first predicted by PITA with the following cutoffs: 1) minimum seed size: 6, and single G:U wobble allowed for seed size of 7 and 8; 2) sites with microRNA-target hybridization energy ΔG_duplex_ score ≤ -15 kcal/mol and combined interaction energy ΔΔG score ≤ -10 kcal/mol; which were further filtered by RNAhybrid: minimum free energy (mfe) ≤ -20 kcal/mol. Target site location within mRNA transcripts (5'-UTR, CDS, or 3'-UTR) was further determined by the annotation available in NCBI database.

## Results and Discussion

### Global view of gender-biased expressed genes in *S*. *japonicum*

Based on the results generated from the use of a microarray with the most comprehensive and informative probe design to date, signal intensities from 4,303 and 6,224 sequences were up-regulated (FC ≥ 2) in male and female adult worms, respectively, which enabled us to retrieve 2,459 (1,344 and 1,115 male and female-biased expressed genes, respectively) potential gender differentially expressed genes from NCBI database (S2 Table). These gene sets further underwent screening with stringent criteria (See Materials and Methods and Table 1). Since alternative splicing and bidirectional transcription are frequent events in the *S*. *japonicum* transcriptome [20,46], these criteria can significantly increase the accuracy but may sacrifice sensitivity of the screening procedure to some degree. The procedure finally led us to identify 685 and 430 mRNA transcripts, and 130 and 86 expressed sequence tags (ESTs), exhibiting gender biased expression in male and female worms, respectively (Table 1 and S3–S6 Tables). In comparison with other reports [10,13,21,49], a relatively small number of RNA transcripts (~12%, 85 male-biased and 51 female-biased) were identified as previously “reported” gender-associated genes (S3 and S4 Tables), thereby allowing us to further explore these novel gender-associated genes in *S*. *japonicum*.

**Table 1:** Screening of gender differentially expressed genes in *S*. *japonicum* from the microarray data and miRNA target sites analysis within the gene sets.

	Male>Female		Female>Male	
Transcripts (number)	mRNA (1094)	EST (250)	mRNA (838)	EST (277)
FC of the mean of the intensity values > = 2 (both forward and reverse probe sets)Mean of signal intensity >100 at least in one gender*t*-test (*p*<0.05)	685	130	430	86
Genes with miRNA target sites	330	55	216	43
Genes without miRNA target sites	355	75	214	48
miRNA target sites	552	95	388	56

**Fig 1:**
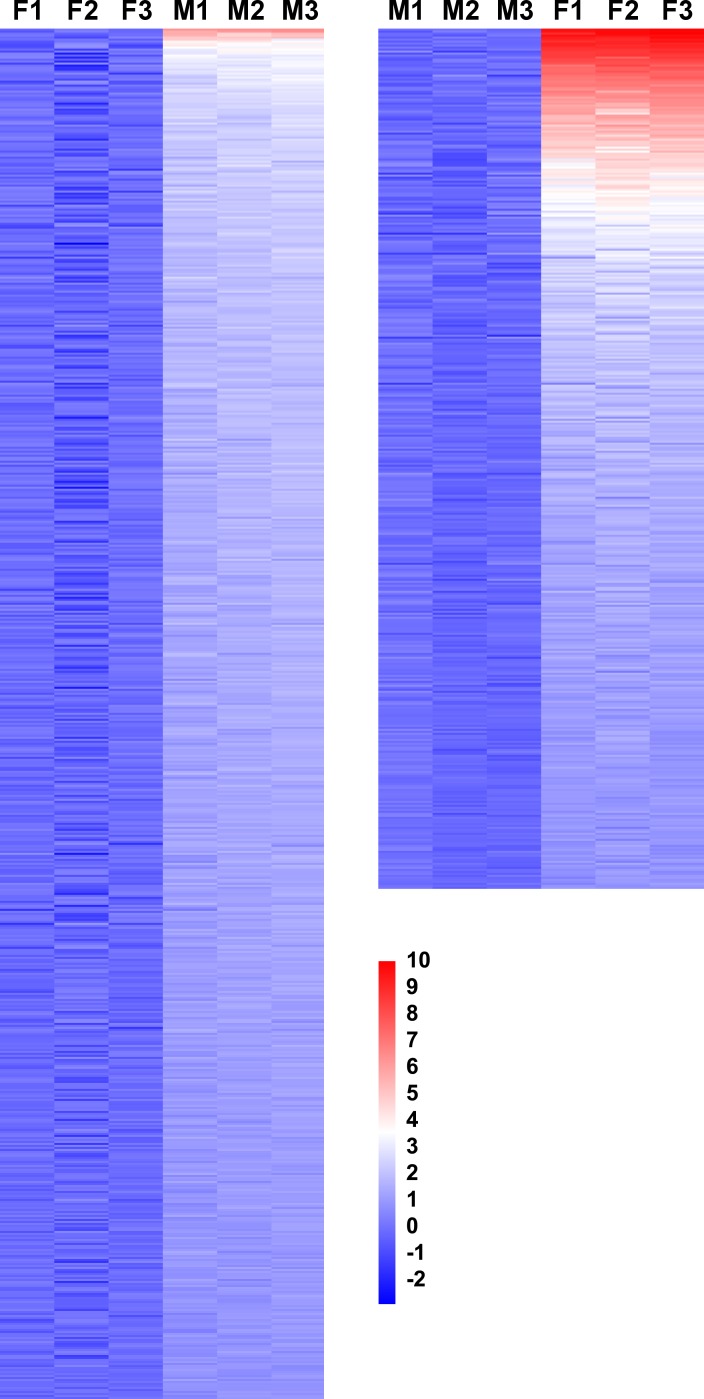
Heatmap for the gender-biased expressed genes determined by probes designed based on genomic and transcriptomic, but not EST sequences. A total of 685 and 430 genes are adult male-biased (left panel) and female-biased (right panel) in their expression, respectively. The presented data are based on the signal intensity of forward sequences. The heatmap was constructed based on the transformed data of log_2_ fold change data. Three biological replicates are presented.

**Fig 2:**
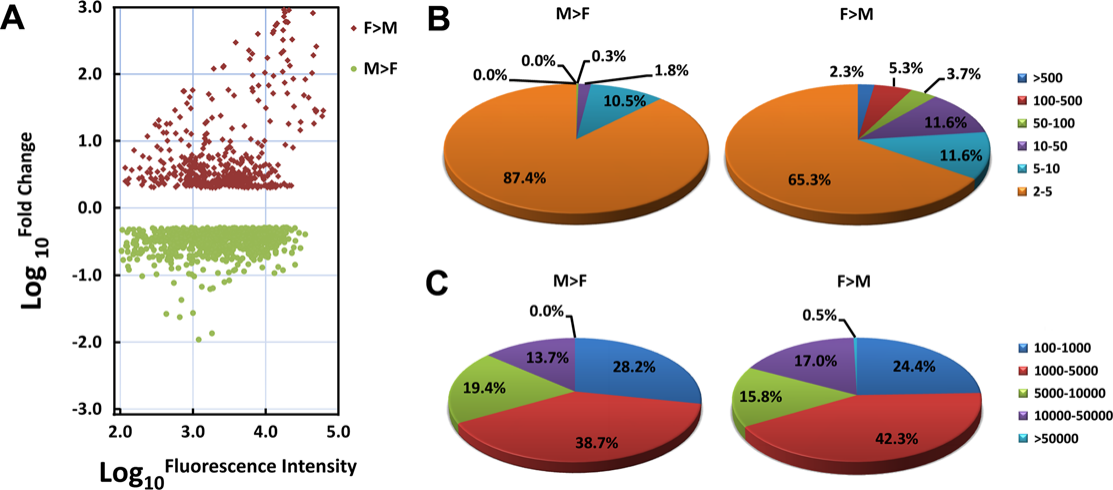
Bias ratio and signal intensity analysis of gender-biased expressed genes. **A**. Scatter plot showing the distribution of the bias ratio for adult male and female-associated genes. The Y-axis corresponds to a log_10_ fold change between adult male and female worms and the X-axis corresponds to the log_10_ fluorescence intensity (enriched in males—green or in females red); **B.** Percentage of genes showing different fluorescence intensities; **C.** Percentage of genes showing different bias ratios.

In general, the number of male-associated genes was higher than female-associated genes, yet more transcripts in female-associated genes exhibit a stronger biased (greater fold change) expression (Fig 1, Fig 2A and 2B). For example, 7% female-associated genes show a strong biased expression (fold change >100) compared with male worms, while none of male-associated genes presented a fold change >100. The percentage of genes showing a high fluorescence intensity (>10,000) in male- and female-biased expressed genes were 13.7%, and 17.5% respectively (Fig 2C), although this parameter may be affected by the GC composition of the 60-mer probes. A similar result was obtained when analysing the gender-biased EST sequences (S2 Fig), but with a low number of genes (about 1/5 compared to the mRNA data). Furthermore, most of these EST sequences were annotated as either unknown or as hypothetical protein (81 (62.30%) and 47 (54.65%) in the adult male- and female-biased ESTs, respectively) (S5 and S6 Tables). This may reflect the fact they are short sequences from the 5'-untranslational regions (UTR) and 3' UTR of mRNA transcripts [6]. More importantly, a comparison of the mRNA and EST data highlights the power of our second generation *S*. *japonicum* DNA microarray in profiling gene expression, since the design of the first generation of *S*. *japonicum* chip was based on EST data only. A subset of these ESTs overlaps with those gender-biased mRNA transcripts; i.e., Aromatic-L-amino-acid decarboxylase, 22.6 kDa tegumental membrane-associated antigen, putative wnt inhibitor frzb2, semaphorin-5B, 16 kDa calcium-binding protein, ancient ubiquitous protein 1, myosin heavy chain, paramyosin, calponin-3 and Annexin A3, listed in both the male-biased mRNA and EST transcripts, whereas TES (Trematode Eggshell Synthesis) domain containing protein, UV excision repair protein RAD23, alanine aminotransferase 2 and DNA replication licensing factor mcm7-A were listed in both the female-biased mRNA and EST transcripts. We then focused on analysing the gender-biased mRNA data further.

### qPCR validation of DNA microarray data

**Fig 3:**
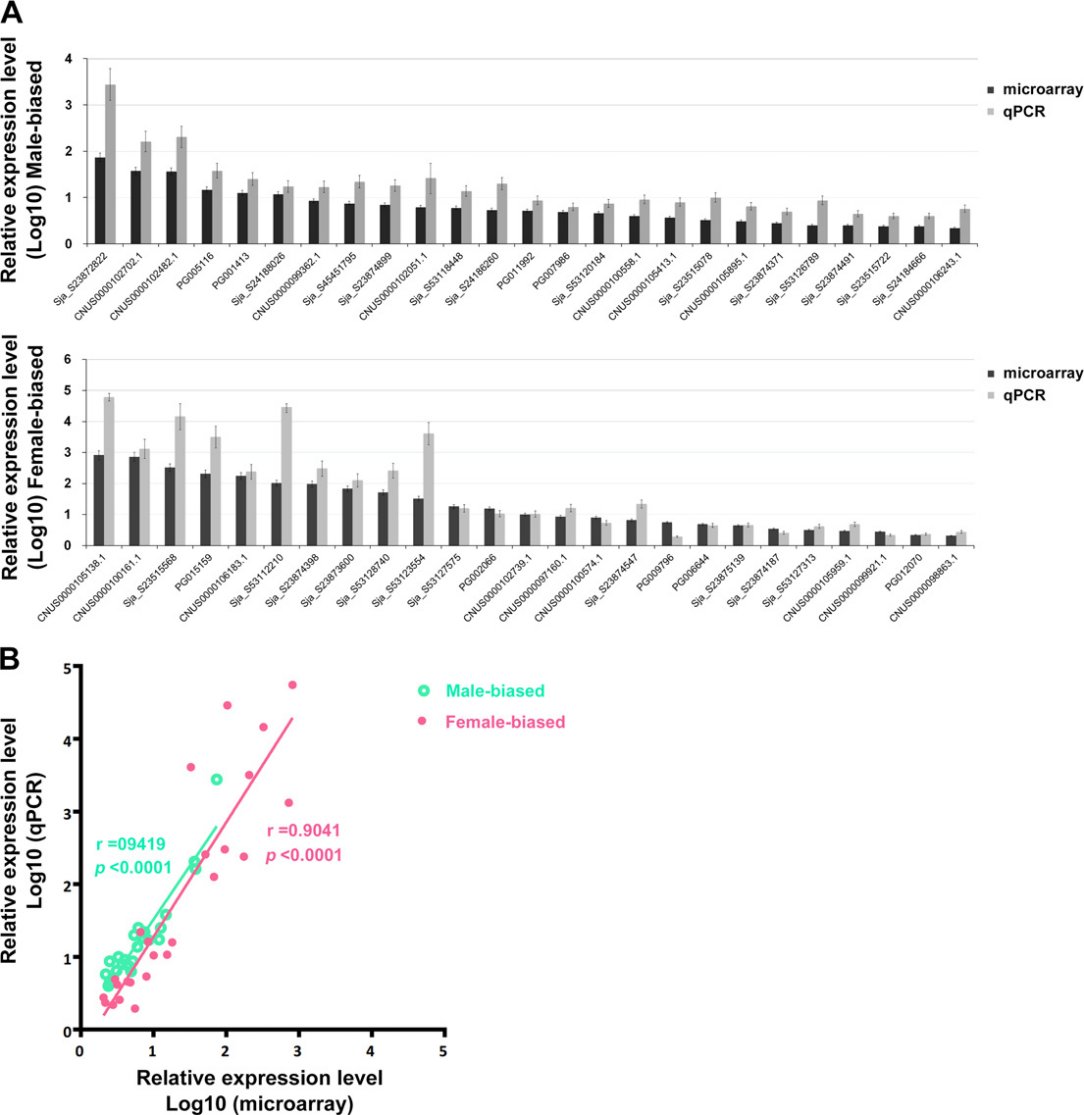
DNA microarray results validated by qPCR assays. **A.** Comparison of DNA microarray and qPCR results of 50 gender-associated genes. Upper panel, 25 adult male-associated genes; Lower panel, 25 adult female-associated genes. **B.** Correlations between the qPCR and microarray results of 50 gender-biased expressed genes were performed using Spearman’s Rho correlation. The log_10_ ratio transformed data are shown.

In order to validate the microarray results, a subset (50, 4.48%) of the gender-differentially expressed genes was selected for validation using qPCR. Generally, the fold changes obtained with the qPCR assay were higher than these obtained by the microarray signals, especially for these extremely biased (high fold change) genes (Fig 3A), which is a phenomenon common in microarray validation experiments [14,50]. Strong correlations were observed between the two methods (for male-biased expressed genes, r = 0.9419, *p*<0.0001; for female-biased expressed genes, r = 0.9041, *p*<0.0001) (Fig 3B). Further, the expression of 10 non-gender-associated genes was also validated by qPCR, which showed good consistency with the DNA microarray data (S3 Fig).

### Putative function prediction by GO analysis

**Fig 4:**
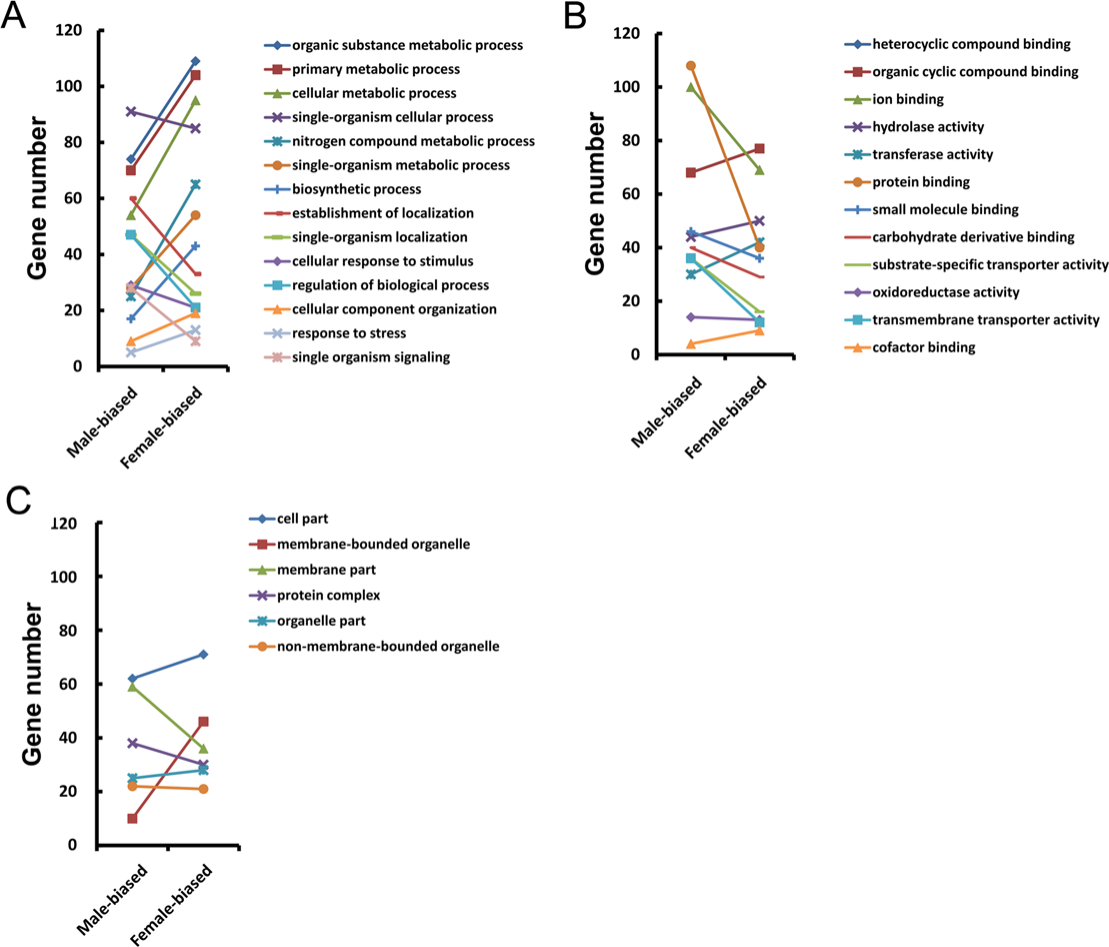
GO distribution for gender-biased expressed genes. The Blast2Go program defined the GO terms into three categories: **A.** biological processes; **B.** molecular functions; **C.** cellular component.

To predict the potential function of these sex-biased genes, different functional categories were further determined by Gene Ontology [51] (Fig 4, S7 and S8 Tables). Of the biological process categories, genes involved metabolic and biosynthetic processes are more active in female worms compared to male worms, indicating that the nutritional acquisition is more crucial for female worms, probably reflective of its status of oviposition which requires abundant nutrition for the laying of thousands of eggs per day per worm pair. This finding was consistent with that of a similar transcriptomic study of *S*. *mansoni* [21], showing that cellular protein modification process, DNA metabolic process and catalytic activity were the top three enriched categories in females. Of the molecular function categories, more genes associated with protein, ion, small molecule and carbohydrate derivative binding; transmembrane and substrate-specific transporter activity were more highly expressed in male *S*. *japonicum* worms, indicating more active host-schistosome interplay (i.e., host ligand-receptor interaction) and energy metabolism than in females. In comparison, assembly, calcium ion binding, protein binding, receptor activity, potassium ion transport and regulation of transmembrane transport were found to be significantly enriched GO categories in *S*. *mansoni* males [21]. In the cellular component categories, gene products localised to membrane regions are more abundant in adult *S*. *japonicum* males, while gene products localised to membrane-bounded organelles more enriched in adult females.

### Analysis of the top 40 gender-biased expressed genes in *S*. *japonicum*

We then further analysed the highest, by fold change, gender-biased expressed genes for each sex (Top 40, Tables 2 and 3). Of the male-specific genes, the majority were previously uncharacterized; thus we inferred their putative functions based on their homology to other species. An example being gamma-crystallin related domain-containing protein (FN317557, ranked 3) which is a Ca^2+^ binding protein, whose biological functions are not fully understood. However, it has been suggested that crystallin in mammals may function in protecting retinal neurons from damage caused by environmental and/or metabolic stress [52]. Aromatic-L-amino-acid decarboxylase (AY812557, Top 5) participates in dopamine and serotonin (5-HT) neurotransmitter synthesis [53]. It has been shown that the expression of its ortholog in the male worms of *S*. *mansoni* was pairing-dependent [54]. Neuroglian (AY811209, Top 10) is a homologue of a L1-type neural adhere molecule in vertebrates. In *Drosophila*, it has been shown that neuroglian is expressed in a variety of embryonic cells, and one of its primary functions is as a central coordinator of synaptic growth [55]. With semaphorin-5B (AY808492, Top 11), its vertebrate equivalents act as positive axonal growth guidance cues as well as functioning in the regulation of synapse morphology [56]. Similarly, spondin-1 (AY812421, Top 22) is an extracellular matrix protein, with its homologue in *C*. *elegans* also acting in axon guidance and fasciculation in motoneurons [57]. The over-expression of these genes in adult males of *S*. *japonicum* collectively indicates that the neuronal activities in this sex are more robust, or play a more prominent biological function, when compared to female schistosomes.

**Table 2:** Top 40 adult male-biased expressed genes in *S*. *japonicum*

NCBI_Nucleotide	NCBI_Protein	Annotation	M:F	*P* value	Accession No.*	E-value
FN313574.1	CAX69308.1	hypothetical protein	91.4	0.000	CCD75631.1	0
AY815593	AAW27325.1	hypothetical protein	73.7	0.003	CCD81827.1	5E-31
FN317557	CAX73288.1	Gamma-crystallin related domain-containing protein	42.2	0.007	XP_012801428.1	2E-28
AY811231.1	AAX27120.2	kelch-like protein 10	37.7	0.006	GAA39156.1	2E-83
AY812557.1	AAX28446.2	Aromatic-L-amino-acid decarboxylase	36.6	0.000	XP_012794006.1Top of FormBottom of Form	1E-112
AY808810.1	AAX24699.2	DUF3091 domain-containing protein	23.3	0.036	AAX25113.2	2E-54
FN319746	CAX75472.1	hypothetical protein	16.1	0.004	AAW27445.1	2E-92
FN317642	CAX73372.1	hypothetical protein	15.5	0.001	CAX75914.1	2E-39
FN314639.1	CAX70372.1	25 kDa integral membrane protein	14.8	0.005	XP_012794735.1	1E-44
AY811209.1	ABA40358.1	Neuroglian	13.0	0.001	XP_012798325.1	2E-75Top of FormBottom of Form
AY808492.1	AAX24381.2	Semaphorin-5B	12.6	0.003	XP_012794855.1	1E-90
AY812167	AAX28056.2	hypothetical protein	11.9	0.008	CCD75512.1	2E-103
AY814934.1	AAW26666.1	hypothetical protein	10.3	0.001	XP_012795660.1	1E-96
AY812625.1	ABA40477.1	Innexin unc-9	10.3	0.007	GAA52696.1	1E-74
AY808991.1	AAX24880.2	hypothetical protein	9.7	0.001	XP_012792735.1Top of FormBottom of Form	3E-82
AY810451.1	AAX26340.2	zinc finger transcription factor Cubitus interruptus	9.6	0.000	CCD59963.1	1E-66
AY915638.1	AAX30859.2	Kinase D-interacting substrate of 220 kDa	9.5	0.001	XP_012792335.1	5E-78
FN314642.1	CAX70375.1	25 kDa integral membrane protein	9.3	0.001	XP_012794735.1	6E-44
AY812312.1	AAX28201.2	hypothetical protein	9.2	0.005	CCD76575.1	4E-18
AY811033.1	AAX26922.2	Forkhead box protein F1	9.0	0.000	CCD60190.1	1E-61
AY808981.1	AAX24870.2	hypothetical protein	8.7	0.003	XP_012794699.1	5E-94
AY812421.1	AAX28310.2	Spondin-1	8.5	0.000	XP_012799285.1	0
FN316932	CAX72663.1	hypothetical protein	8.4	0.001	NA	NA
AY809053.1	AAX24942.2	hypothetical protein	8.3	0.014	CCD79001.1	1E-71
AY809579.1	AAX25468.2	hypothetical protein	8.2	0.015	CCD76427.1	4E-22
FN317889	CAX73618.1	Glutamine-rich protein 2	8.2	0.015	XP_012795786.1	0
FN320192	CAX75918.1	hypothetical protein	7.9	0.007	CAX75914.1	4E-123
AY223389.1	AAP06426.1	Nebulette	7.8	0.020	CCD79737.1Top of FormBottom of Form	1E-154
AY815479.1	AAW27211.1	hypothetical protein	7.6	0.000	CCD82965.1	3E-153
EZ000179	ACE06959.1	Nebulette	7.5	0.004	CCD79737.1	5E-147
AY810562.1	CAX75588.1	Peptidase M8, leishmanolysin domain-containing protein	7.4	0.000	CCD60061.1	4E-127
AY809198.1	AAX25087.2	hypothetical protein	7.1	0.002	CAZ33468.1	7E-29
AY808957	AAX24846.2	hypothetical protein	7.0	0.003	XP_012797249.1	7E-98
AY813515	AAW25247.1	venom allergen-like (VAL) 6 protein	7.0	0.000	CCD74796.1	0
AY810798	AAX26687.2	LIM and senescent cell antigen-like-containing domain protein 1	6.9	0.000	XP_012797616.1	0
AY809721.1	AAX25610.2	hypothetical protein	6.8	0.001	XP_012794055.1	8E-113
AY812926.1	AAW24658.1	hypothetical protein	6.6	0.000	CCD78656.1	3E-49
AY812127.1	AAX28016.2	putative protein serine/threonine kinase	6.6	0.007	CCD77604.1	2E-83
AY809045.1	AAX24934.2	Nephrin	6.2	0.001	XP_012799122.1	2E-116
AY809011.1	AAX24900.2	putative wnt inhibitor frzb2	6.2	0.000	CCD79605.1	3E-76

* Accession number of the closest homologue

**Table 3:** Top 40 adult female-biased expressed genes in *S*. *japonicum*.

NCBI_Nucleotide	NCBI_Protein	Annotation	F:M	*P* value	Accession No.*	E-value
AY813405	AAW25137.1	Trematode Eggshell Synthesis domain containing protein	919.1	0.000	CCD59010.1	4E-33
FN314999	CAX70731.1	Trematode Eggshell Synthesis domain containing protein	904.4	0.000	CCD59010.1	9E-47
AY814142.1	AAW25874.1	FAM75 family member	832.9	0.009	XP_012798449.1	7E-46
FN314868.1	CAX70600.1	Asparagine-rich antigen Pfa35-2	818.2	0.000	CAX70601.1	0
AY812810.1	AAW24542.1	Histidine-rich glycoprotein precursor	726.6	0.001	CAX69384.1	0
FN317103	CAX72834.1	hypothetical protein	606.9	0.000	AAX27197.2	1E-141
AY813556.1	AAW25288.1	Trematode Eggshell Synthesis domain containing protein	606.6	0.004	XP_012797543.1Top of FormBottom of Form	3E-84
FN313912	CAX69646.1	Trematode Eggshell Synthesis domain containing protein	595.9	0.000	CCD59010.1	1E-45
FN314997	AAW27224.1	Trematode Eggshell Synthesis domain containing protein	571.6	0.000	CCD59010.1	3E-42
AY811322.1	AAX27211.2	UV excision repair protein RAD23	531.2	0.000	CCD82179.1	3E-57
AY815518	AAW27250.1	Trematode Eggshell Synthesis domain containing protein	453.5	0.000	CCD59010.1	5E-42
AY815264.1	AAW26996.1	Tyrosinase	443.7	0.001	AAP93838.1	0
FN313788.1	CAX69522.1	Beta/gamma crystallin	410.8	0.000	CCD74684.1	3E-61
FN317243	CAX72974.1	Splicing factor U2AF 65 kDa subunit	399.2	0.000	CAZ29648.1	0
AY812315.1	AAX28204.2	hypothetical protein	329.4	0.008	XP_012792673.1	2E-22
AY222962	AAP05974.1	tetraspanin, putative	324.8	0.000	CCD58628.1	3E-142
FN327074	CAX82798.1	Large neutral amino acids transporter small subunit 2	297.1	0.000	CCD80585.1	0
EZ000096	ACE06876.1	eggshell protein, chorion	259.1	0.000	CCD59975.1	4E-51
FN319535	AAP06415.1	Annexin-B12	226.3	0.019	CCD80864.1	0
FN316055.1	CAX71782.1	Extracellular superoxide dismutase [Cu-Zn]	220.9	0.001	XP_012794484.1	4E-103
AY812904.1	AAW24636.1	tyrosinase 2	206.1	0.001	AAW21822.1	0
FN319742.1	CAX75468.1	Globin-3	201.3	0.002	XP_012795763.1	6E-82
FN315504.1	AAW25976.1	Trematode Eggshell Synthesis domain containing protein	191.2	0.000	XP_012794933.1	1E-59
FN317561.1	CAX73292.1	trypsin-like serine and cysteine peptidase domain containing protein	174.8	0.000	XP_012792462.1	3E-114
FN313659.1	CAX69393.1	Poly(rC)-binding protein 3 (Alpha-CP3)	167.4	0.005	CCD79374.1	1E-117
FN314903.1	CAX70634.1	hypothetical protein	160.3	0.000	CCD77371.1	2E-38
FN313935.1	CAX69669.1	hypothetical protein	133.5	0.000	XP_012794935.1	4E-09
FN317391.1	CAX73122.1	Histone H2A	132.3	0.000	XP_012795189.1	1E-80
EZ000032.1	AAP06288.1	cell division cycle 20 (fizzy)-related	127.1	0.001	CCD82273.1	7E-168
AY812388.1	AAX28277.2	putative propionyl-CoA carboxylase alpha subunit	120.5	0.000	CCD74939.1	1E-32
AY815418	AAW27150.1	Female-specific protein 800	108.7	0.001	CCD59009.1	1E-27
AY808975.1	AAX24864.2	60S ribosomal protein L19, putative	105.1	0.018	CCD58962.1	2E-43
FN330801	CAX83018.1	Stress protein DDR48 (DNA damage-responsive protein 48)	103.9	0.000	AAA29908.1	5E-69
AY814016	AAW25748.1	Trypsin-like serine protease	95.8	0.000	XP_012793577.1	4E-160
AY813874	AAW25606.1	CLECT Superfamily member	94.1	0.004	CCD60786.1	4E-104
FN313715.1	CAX69449.1	Trematode Eggshell Synthesis domain containing protein	85.1	0.000	CCD59010.1	2E-34
AY222885	AAP05897.1	Stress protein DDR48 (DNA damage-responsive protein 48)	83.5	0.001	CCD59978.1	3E-64
AY812649.1	AAX28538.2	CLECT Superfamily member	74.5	0.005	CCD59786.1	1E-87
AY814814	AAW26546.1	CLECT Superfamily member	67.8	0.014	XP_012793832.1	2E-137
FN313682.1	CAX69416.1	putative reticulocalbin	67.5	0.000	CCD80508.1	4E-173

* Accession number of the closest homologue

In addition, the Top 4 gene (AY811231) encodes a kelch-like protein 10, which may be related to spermiogenesis, since its vertebrate equivalents are testis-specific, and it has been suggested to participate in protein ubiquitination and subsequent proteasomal degradation during spermiogenesis [58]. Another interesting gene highly expressed in males is the zinc finger transcription factor Cubitus interruptus (AY810451, Top 16), whose counterpart in *Drosophila* can act as a mediator of hedgehog signal transduction to facilitate normal osteoblast differentiation via regulating the expression of the decapentaplegic gene [59], the ortholog of mammalian bone morphogenetic protein (BMP) 2. A homologue of BMP has been characterized in *S*. *mansoni*, which is also more abundant in male parasites, but its localization remains elusive [60]. Furthermore, nebulette (AY223389, Top 31) is a nebulin-like protein, its vertebrate counterparts interacting with a number of skeletal proteins such as actin, tropomyosin, alpha-actinin, Xin, and XIRP2 [61], and function in regulating the assembly and lengths of thin filaments in skeletal muscle [62], indicating this protein is related to the motility of the parasite. Previous research in vertebrates has indicated that nephrin is a structural component of the slit diaphragm [63]. However, a recent study showed that the nephrin homologue in planarians is probably expressed in neoblasts, but not in flame cells and neurons [64]. It would be of value to determine the molecular localization of nephrin in schistosomes which may provide further clues to its precise cellular function.

A number of trematode eggshell synthesis (TES) domain containing proteins, an asparagine-rich antigen Pfa35-2, a histidine-rich glycoprotein, an extracellular superoxide dismutase (Ex-SOD), a female-specific 800 protein, two distinct of tyrosinase homologues are listed in the top 40 female-associated genes (Table 3). A finding consistent with previous studies on schistosomes, with the potential molecular functions for some of these genes having been suggested [10,11,15,16,21,49,65]. The data presented here thus show the consistency of high-throughput gene profiling technologies in confirming these highly differentially expressed genes. Intriguingly, it has been shown that DNA vaccination with one of these genes, Cu-Zn SOD, induced a high reduction of worm burden in mice infected with *S*. *mansoni* [66]. Further, it is noteworthy to consider some of the novel genes listed in Table 3, which exhibit a variety of molecular functions. For example, the top 10 gene encodes a UV excision repair protein, RAD23, which is involved in nucleotide excision repair (NER). This may be linked to the extensive DNA damage caused by antioxidants released during the detoxification of hemoglobin byproducts in females, which is discussed further below. The gene encoding a large neutral amino acids transporter small subunit 2 (Top 17) was expressed specifically in females, which likely reflects their special physiological status requiring a considerable amount of large neutral amino acids [67]. Furthermore, poly(rC)-binding protein 3 (Alpha-CP3) (Top 25) is a member of the KH-domain containing protein, which binds to RNA transcripts via a C-rich pyrimidine region, and further mediates post-transcriptional regulatory activities. Previously, miRNAs, another post-transcriptional regulatory factor, have also been shown to exhibit sex-biased expression in schistosomes [26,30]. This observation indicates that both Alpha-CP3 and miRNAs are responsible for post-transcriptional gene regulation in *S*. *japonicum* by targeting different gene sites.

### Genes encoding cytoskeleton and motor proteins are more up-regulated in male adult worms

In general, previous studies on schistosomes have shown consistently that genes encoding cytoskeleton and motor proteins (i.e., actin, titin, alpha-actinin, dynein light chain, myosin heavy chain, paramyosin, tropomyosin, fimbrin, and troponins) are up-regulated in male worms [10,13,15,16,20,49]. This was also reflected by enriched GO analysis of *S*. *mansoni* adult worms showing that microtubule, microtubule-based process and troponins complex were significantly enriched GO categories in males [21]. These data thus indicate a role for males in the physical support of females, which enables the latter to save energy and to focus on nutrient acquisition and egg-laying. More cytoskeleton genes, such as microtubule-associated protein 2, actin related protein 2/3 complex, tensin, laminin were identified in this study as a result of using our comprehensive probe design. In addition, other genes encoding cytoskeleton related proteins, such as PDZ-, multiple PDZ- or PDZ and LIM domain-containing proteins (FN317962, AY810295, AY811780, AY812842, AY815664, AY808539, AY812903 and AY814003) (S3 Table), which contribute to the formation and maintenance of cell complex scaffolding were highly expressed in adult males. The majority of these genes were 2–4 fold more highly expressed in male worms compared with females, which supports the notion that male worms render physical support to females to facilitate their migration against the blood flow from the portal liver sites to the smaller mesenteric circulation where they lay their eggs [11]. Further, members of the PDZ domain-containing protein, such as GIPC3 and Scribble, have been suggested as potential drug targets based on non-canonical protein interaction, though they do not display a sex-biased expression [68,69].

### Genes involved in neuronal activities are more up-regulated in male adult worms

Motor activity in schistosomes is closely controlled by the neuronal system. In addition to the male-biased transcripts related to neurotransmitter synthesis, synapse growth and axon development, discussed earlier, additional genes involved in neuronal activities were also found enriched in male worms, including ionotropic glutamate receptor (AY815670), neuron navigator 3 (AY808520), neurogenic locus notch protein-like protein (AY809231), excitatory amino acid transporter (AY810837), neuronal calcium sensor 2 (FN317645), and synaptic vesicle membrane protein VAT-1-like protein (AY811071) (S3 Table). The data reinforce the active neuronal activities in male parasites, which could be linked to the fact that the adult male worms are directly exposed to the cardiovascular system and need to monitor and respond to environmental cues from the host [20]. One should be aware that schistosome neuronal system is not only responsible for motor activity, but also plays an essential role in a wide variety of biological processes, such as cercarial penetration, blood feeding and digestion, waste disposal, reproductive activities, and egg excretion [70], highlighting the significance of the neuronal signaling pathways for parasite survival. Several neuronal receptors, such as the glutamate receptor (SmGluR) [71], serotonin receptor (Sm5HTR) and G protein-coupled acetylcholine receptor, have been suggested as potential targets for novel drug development against *S*. *mansoni* [72,73]. Further, it has been shown that two glutamate receptor genes (GRIN1 and NMDA receptor) were up-regulated in paired males treated with PZQ [74]. The expressed products of the neuronal pathway-related genes identified here may represent alternative targets for drug development against the schistosome parasites.

### Genes involved in amino acid metabolism, nucleotide biosynthesis and gluconeogenesis are more up-regulated in female adult worms

As indicated by the GO analysis, metabolic and biosynthetic processes are more vigorous in female worms (Fig 4A). This is supported by the fact that genes encoding enzymes participating in a variety of metabolic pathways were actively transcribed within this sex. Several members of the venom allergen-like (VAL) family (i.e., VAL 27 (FN318592) and 28 (AY815621)) were also significantly more highly expressed in female parasites. Previously, esophageal secreted proteins encoded by micro exon gene (MEG) 4.1, 4.2, and 14 and VAL-7 in *S*. *mansoni* have been shown to play a vital role in erythrocyte lysis and tethering and killing of leucocytes [75]. Recently, SjMEGs 4.1, 8.2, 9, 11 and VAL-7 have been suggested as potential targets of the self-cure process based on observations with the *Rhesus Macaques* animal model [76]. It would be interesting to determine the localization and function of VAL-27 and 28, which may play a specific role in the biology of female parasites. Increased amino acid metabolism could be expected in females based on the observation that genes involved in amino acid transport (i.e., large neutral amino acids transporter (FN327074) and L-amino acid transporter (FN313722)) and amino acid metabolism (i.e., putative L-asparaginase (AY814032), gamma-glutamylcyclotransferase (AY814775), alanine aminotransferase (AY915267) and aspartate-ammonia ligase (FN326707)) were up-regulated in this sex (S4 Table). In addition, genes related to nucleotide biosynthesis (i.e., adenylosuccinate synthetise (AY816019), ribonucleoside-diphosphate reductase subunit M1 (FN330781) and hypoxanthine-guanine phosphoribosyltransferase (AY915002)) were enriched in adult females, emphasising the increased DNA synthesis is evident during vitellocyte differentiation in female parasites. In respect to energy metabolism, it has been shown that, in schistosomes, two typical facilitated diffusion glucose transporter proteins 1 (GTP1) and 4 (GTP4) are responsible for transporting glucose from the exterior to the inside of worms [77,78]. However, no sexually biased expression was observed for these genes in the current study, in contrast to genes encoding enzymes involved in gluconeogenesis, phosphoenolpyruvate carboxykinase (AY813371) and fructose-1,6-bisphosphatase 1 (FN318294), showing a 2.2 and 2.8 fold up-regulation, respectively, in female than in male worms. These observations indicate that the female parasite may have a relatively increased ability to generate glucose from non-carbohydrate carbon substrates, and this characteristic may need to be considered when targeting glucose metabolism for potential vaccine candidates.

### Genes involved in cell cycle processes are more up-regulated in female adult worms

A female worm must pair with a male to become completely sexually mature, when the reproductive organs, mainly the ovary and the vitelline glands, undergo terminal differentiation. The vitellarium contributes two thirds of the mature female body volume, and a select number of cells within this structure undergo stage 2 and 3 differentiation, and further terminal differentiation [19,79]. This transcriptional basis of this phenomenon was further supported in our study by observation that genes associated with cell differentiation were highly expressed in female worms (S4 Table). Examples of such genes include G2/mitotic-specific cyclin-B3 (AY809873), Polo-like kinase (FN317236), abnormal spindle-like microcephaly-associated protein (AY812148), cyclin-dependent kinase 1 (AY815214), cell division cycle 20 (fizzy)-related protein (AY223249), Cyclin-T2 (FN317410) and regulator of chromosome condensation (AY810273). Further examples include those associated with cell cycle arrest in response to DNA damage or spindle abnormalities, including cell cycle checkpoint control protein RAD9B (AY812096), mitotic spindle assembly checkpoint protein MAD2A (AY814258), checkpoint protein HUS1 (AY813370), serine/threonine-protein kinase chk2 (FN313971), mitotic checkpoint serine/threonine-protein kinase BUB1 beta (AY808857). It has been shown that apoptosis is an important cellular process in schistosomes [80]. The activities of caspase-3 and -7, both central proteolytic enzymes involved in this process, were active across different developmental stages of *S*. *japonicum*, with a peak expression in the schistosomula 14 days p.i. [80]. Here, the expression of apoptosis-related genes, caspase 7 (AY813428), programmed cell death protein 2 (AY814013) and 4 (AY814519), as well as serine/threonine-protein kinase pim-1 (FN317924), was more extensive in female worms, which may represent an instinct response to get rid of damaged cells.

### Genes involved in DNA synthesis and genome fidelity and stability are more up-regulated in female adult worms

Use of an autoradiographic method has shown that the pairing of male and female parasites impacts on the DNA synthesis in females, but not in males [38]. DNA synthesis may present an important cellular process as a consequence of vitellocyte differentiation. Here, we found a set of genes involved in DNA replication processes, were preferentially expressed in female worms (S4 Table). These included DNA replication licensing factor mcm2 (AY815400), mcm4 (AY914892), and mcm7-A (AY815974), DNA polymerase alpha subunit B (AY811018), Origin recognition complex subunit 4 (AY812655), DNA replication factor Cdt1 (FN313910) and DNA replication complex GINS protein PSF2 (AY815313), were all up-regulated in females. In addition, transcripts for a number of DNA damage repair related genes (i.e., UV excision repair protein RAD23 (AY811322), DNA repair protein RAD51 (AY812723), DNA polymerase epsilon subunit 2 (AY815035), and DNA mismatch repair protein msh2 (AY814227) and msh6 (AY811972)) as well as genes encoding a chromosome transmission fidelity protein (AY810243) and a mini-chromosome maintenance complex-binding protein (AY810626) were also enriched in female parasites. These observations potentially reflect the need for repairing DNA damage caused by oxygen radicals released during the process of hemoglobin digestion, and the guarantee of chromosomal fidelity during vitellocyte mitosis and/or egg embryonic development. Furthermore, the gene encoding Argonaute 2, a protein which binds small interfering RNA, was found over-expressed in adult females, which is consistent with the results of a previous study [46]. The expression of the Ago2 ortholog in *S*. *mansoni* was observed predominantly in the gonads (particularly in the posterior ovary) [81], and SjAgo2 has been shown to play a vital role in germline cell maintenance via suppression of the activity of transposable elements (TEs) [46].

### Genes involved in glycosylation are more up-regulated in female adult worms

Glycosylation in schistosomes is a complex process which plays an essential role in host-pathogen interplay, particularly in terms of immune evasion and modulation [82]. A comprehensive glycomic analysis has revealed that the dominant N-glycans structure dynamically changes during the development of *S mansoni*. For example, N-glycans with Galβ1–4 (Fucα1–3) GlcNAc (LeX) and core-xylose motifs are abundant in cercariae, but are lost rapidly after entry to the mammalian host, while GalNAcβ1-4GlcNAc (LDN)-motifs gradually became predominant during the transition of schistosomula to adult worms. Further, fucosylated motif-enriched N-glycans are presented during egg development [83]. Also, it has been shown that tri-antennary type glycans are predominant in adult females compared with adult males [84]. In the current study, a number of enzymes involved in N-glycan precursor synthesis (putative dolichyl pyrophosphate Glc1Man9GlcNAc2 alpha-1,3-glucosyltransferase (FN313664) and Dol-P-Man:Man(5)GlcNAc(2)-PP-Dol alpha-1,3-mannosyltransferase (AY814785)), trimming (putative mannosyl-oligosaccharide glucosidase (AY809831) and mannosyl-oligosaccharide 1,2-alpha-mannosidase IA (AY915059)) and extension (glycosyltransferase 25 family member (AY810454)) as well as O-linked oligosaccharide biosynthesis (polypeptide GalNAc transferase 6 (FN318098)), were more readily up-regulated in females, suggesting that glycosylation is relatively more active in females and that some specific N-glycan structures are more predominant in this sex. One exception is beta-1,4-galactosyltransferase (AY810750), which was more highly expressed in adult males, which seems to conflict with the situation reported in *S*. *mansoni* where N-glycans enriched in females are frequently terminated with a Galβ1-4GlcNAc motif [84], a process that requires a high beta-1,4-galactosyltransferase activity. However, this may be explained by the fact that multiple beta-1,4-galactosyltransferase isoforms occur in schistosomes [8].

### Hypothetical genes in male and female adult worms

Within those gender-associated genes, a wide array of genes was annotated as hypothetical protein (155 (22.6%) and 95 (22.1%) in adult male- and female-biased expressed genes, respectively). These hypothetical genes may encode schistosome-specific proteins that lack homologous domains with other species, but limited attention has been paid on this gene set. Further research on these genes and their expressed products may further the discovery of new vaccine candidates and drug targets.

### miRNA target prediction against gender-biased expressed genes

miRNA profiles have been established across the different developmental stages and different sexes of *S*. *japonicum* and *S*. *mansoni*, and the potential function for some miRNAs have been suggested based on the profiling data, but their precise roles, such as how they regulate potential targets, remain elusive. Target prediction is an important pipeline in order to learn about the function of miRNAs. Previous miRNA target prediction has been carried out on schistosomes mainly within the 3' UTR of mRNAs [29,85]. However, some studies have shown that the target sites are not limited to the 3' UTR and can be located within the CDS and even the 5' UTR [86,87]. In addition to canonical target sites, non-canonical sites (i.e., “non-seed” sites [88,89]) have widespread biological functions, which undoubtedly increases prediction complexity. Here, we have focused on the mechanism whereby miRNAs can potentially regulate the expression of gender-associated genes.

**Fig 5:**
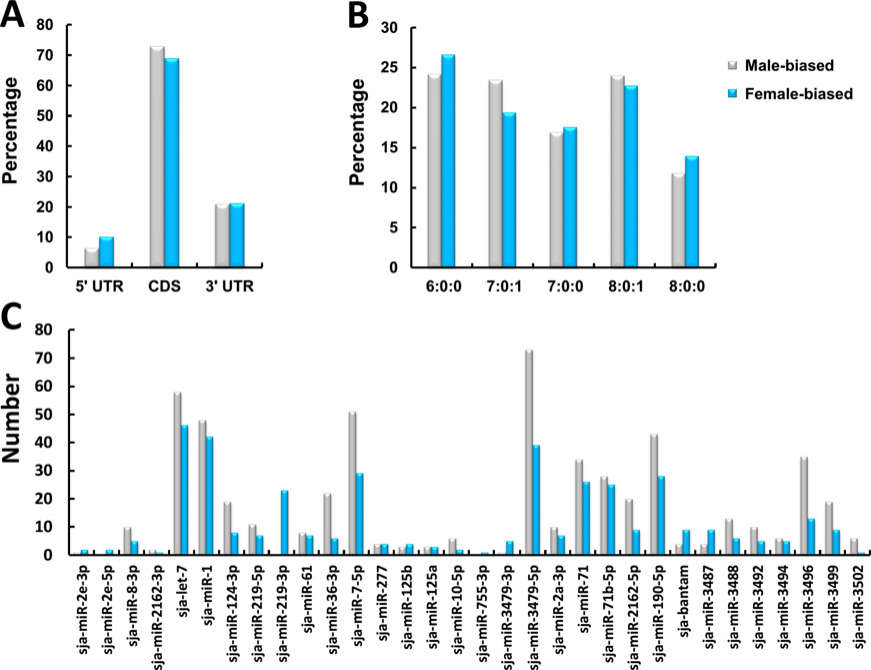
Analysis of putative miRNA target sites within gender-biased expressed genes. **A.** Distribution of miRNA target sites within different mRNA regions (5'-UTR, CDS and 3'-UTR); **B.** Distribution of miRNA target sites with different seed types. The "X:Y:Z" notation used for describing the seed represents the size of the seed (X), the number of mismatches (Y) and the number of G:U wobble pairs (Z); **C.** Target site number for individual miRNA.

Combining the algorithms of PITA [47] and RNAhybrid [48], putative miRNA target sites were predicted against the full length mRNA transcripts that exhibited gender differential expression. Half of these were predicted to contain miRNA target sites (Table 1, S9 and S10 Tables). On average, 1.6 putative miRNA target sites were predicted per individual gene, with most sites located within the CDS (~70%) of both male and female biased expressed genes (Fig 5A). Work with HEK293 cells has shown that within the exonic crosslink-centered regions, 50% of sites correspond to the CDS compared with 46% to 3' UTRs [90]. In another study, it was shown that 41% and 40% miRNA binding sites were located in the 3' UTRs and CDS, respectively, in the human brain [91]. The over-concentration of binding sites in the CDS reported here may have been caused by the fact that the 3' UTR may be fractured in a group of *S*. *japonicum* mRNA transcripts (only about 10% gender-associated mRNA transcripts have a complete 3' UTR based on the presence of a poly(A) tail).The seed type plays has an important impact on miRNA regulatory function [92]. Here in our analysis, no mismatch was allowed in the seed site, and a single G:U wobble was only allowed for seed sizes of 7 and 8. The percentage of target sites was gradually decreased in size type of 6:0:0, 7:0:0 to 8:0:0, while the percentage of target sites was similar for seed types of 7:0:1 and 8:0:1 (Fig 5B). Individually, sja-let-7, sja-miR-1 sja-miR-7-5p, sja-miR-3479-5p sja-miR-190-5p, sja-miR-71 and sja-miR-71b-5p have the most putative sites within the sex-biased expressed genes (Fig 5C) of which sja-let-7, sja-miR-1 sja-miR-7-5p are male-biased miRNAs, while sja-miR-71b-5p is female-biased [26]. In contrast, few target sites have been predicted for sja-miR-125b and sja-bantam, two miRNAs abundantly expressed in male and female worms, respectively, indicating that they may regulate non-gender-associated genes.

### Conclusions

In this study, we present the most comprehensive transcriptomic perspective on sex-expressed genes in *S*. *japonicum*, which sheds further light on key biological and physiological features of the male and female parasites. Furthermore, we present a global view on how miRNAs potentially modulate the expression of gender-associated genes via miRNA target sites prediction. This study provides novel insights on schistosome conjugal biology, which may help in the discovery of new anti-fecundity vaccine candidates and drug targets against this persistent pathogen.

## Supporting Information

S1 Fig Melt curves for each gene validated by qRT-PCR. (PNG)

S2 Fig Heatmap for the gender-biased expressed genes determined by probes designed based on EST sequences. Left panel, adult male-biased genes; right panel, adult female-biased genes. The data are presented based on the signal intensity of forward EST sequences. The heatmap was constructed based on the transformed log_2_ fold change data. Three biological replicates are presented.
(TIFF)

S3 Fig Comparison of DNA microarray and qPCR results for 10 genes that are non-differentially expressed between genders. Female/male fold changes are presented.
(PNG)

S1 Table Primer sets used for qPCR validation. (XLSX)

S2 Table Initial retrieval of gender-biased expressed genes in *S*. *japonicum* from NCBI database based on the DNA microarray data. (XLSX)

S3 Table Detailed information for adult male-biased expressed genes (mRNA data, forward probe). (XLSX)

S4 Table Detailed information for adult female-biased expressed genes (mRNA data, forward probe). (XLSX)

S5 Table Detailed information for adult male-biased expressed genes (EST data, forward probe). (XLSX)

S6 Table Detailed information for adult female-biased expressed genes (EST data, forward probe). (XLSX)

S7 Table Detailed GO annotation for adult male-biased expressed genes. (XLSX)

S8 Table Detailed GO annotation for adult female-biased expressed genes. (XLSX)

S9 Table Putative miRNA target sites within adult male-biased expressed genes. (XLSX)

S10 Table Putative miRNA target sites within adult female-biased expressed genes. (XLSX)
