# Supplementary figures and images for: Comprehensive Transcriptome Analysis of Sex-Biased Expressed Genes Reveals Discrete Biological and Physiological Features of Male and Female Schistosoma japonicum
Source: PLoS Negl Trop Dis. 2016 Apr 29;10(4):e0004684. doi: 10.1371/journal.pntd.0004684 (PMC4851400; doi:10.1371/journal.pntd.0004684)

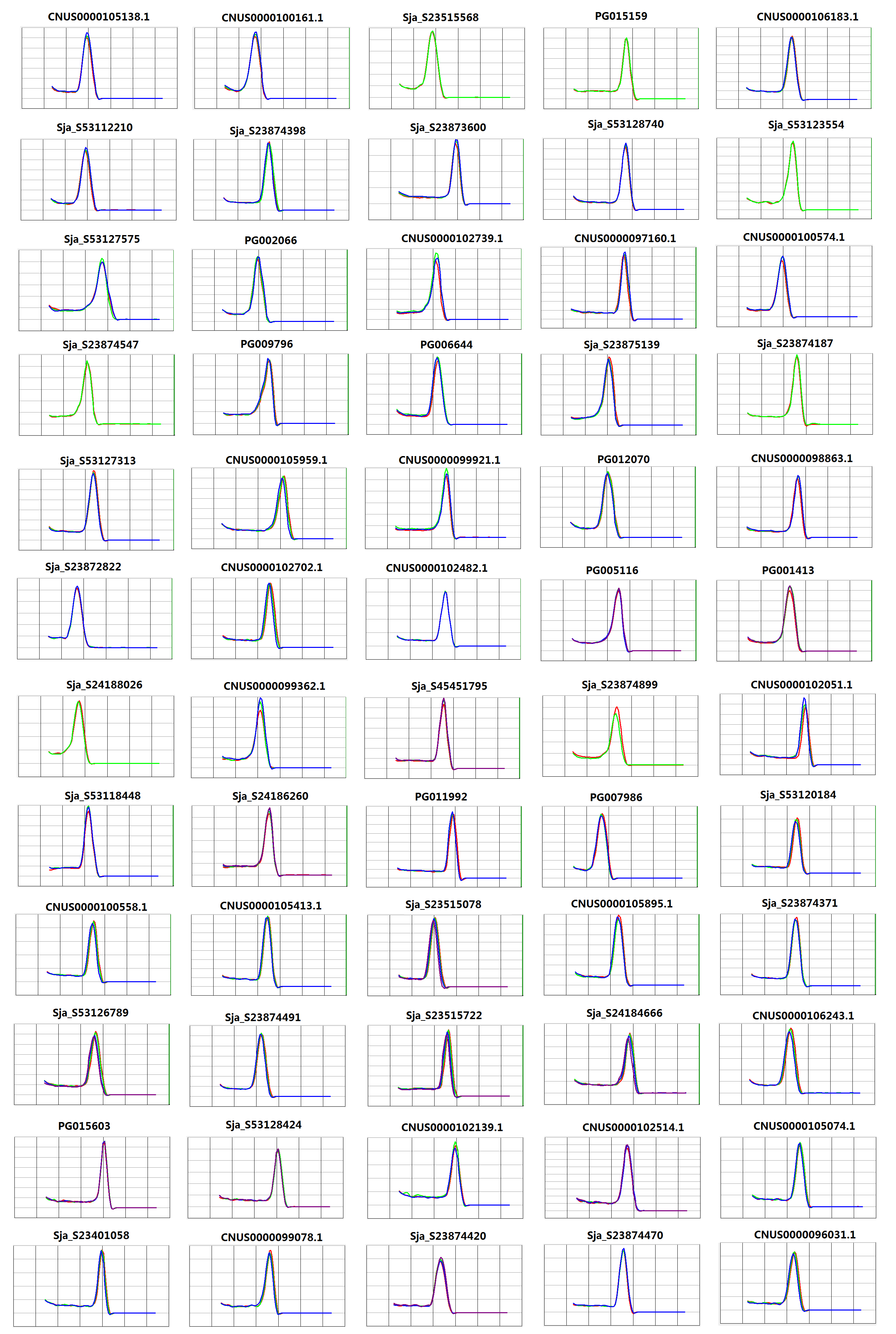

Supplement: S1 Fig — (PNG) [file pntd.0004684.s001.png]

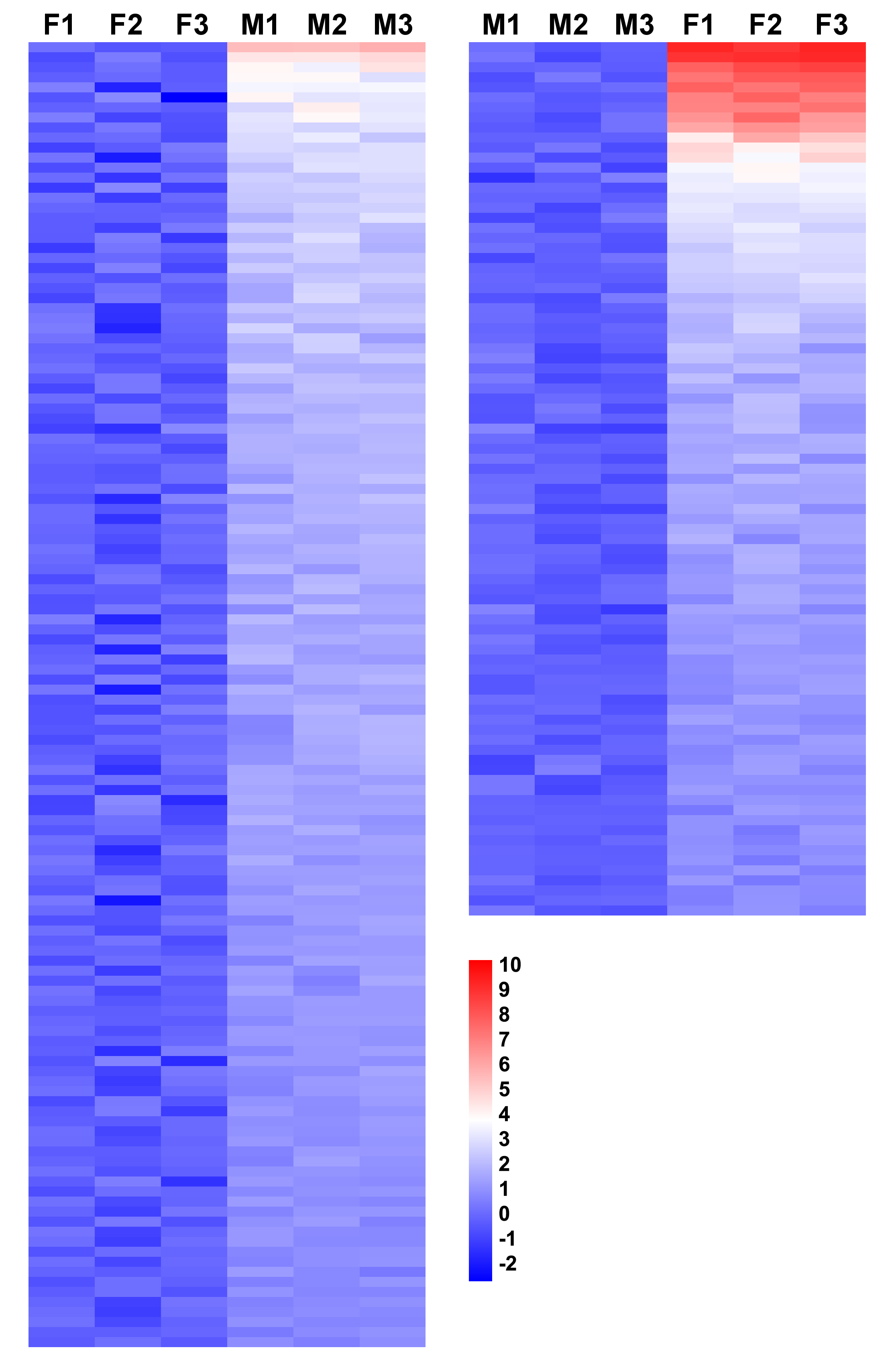

Supplement: S2 Fig — Left panel, adult male-biased genes; right panel, adult female-biased genes. The data are presented based on the signal intensity of forward EST sequences. The heatmap was constructed based on the transformed log2 fold change data. Three biological replicates are presented. (TIFF) [file pntd.0004684.s002.tiff]

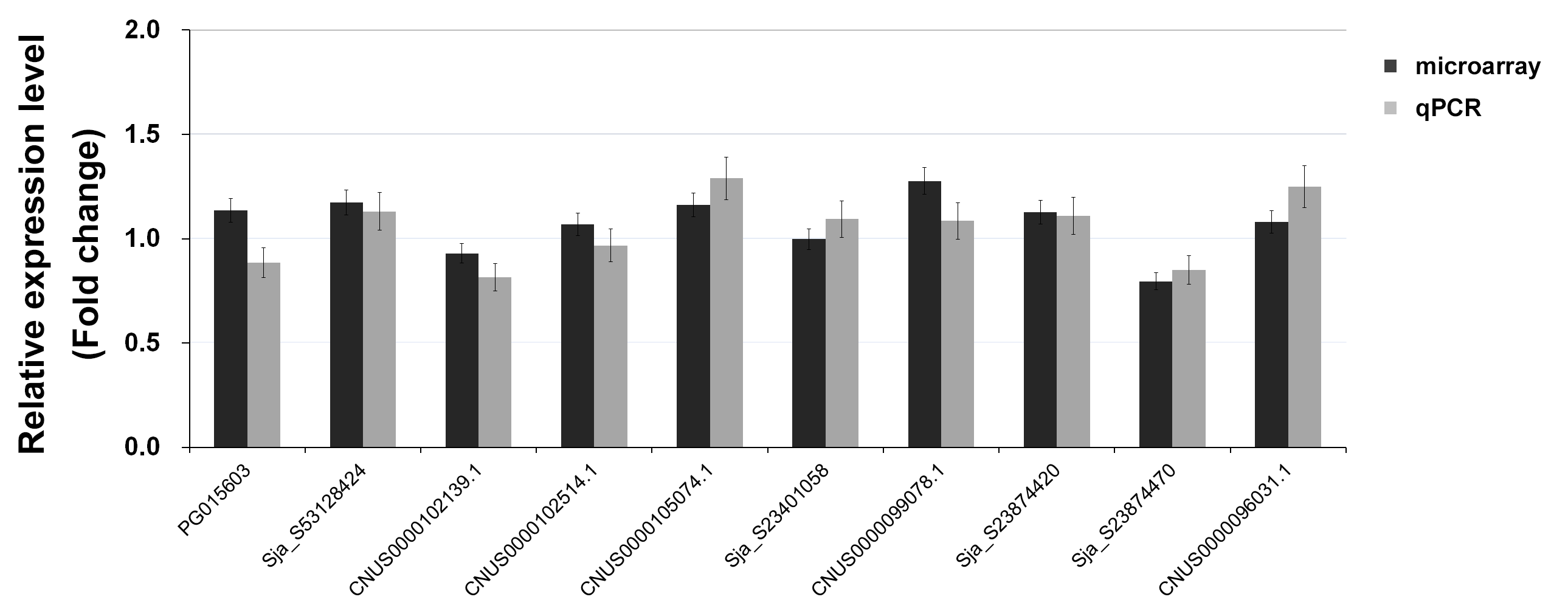

Supplement: S3 Fig — Female/male fold changes are presented. (PNG) [file pntd.0004684.s003.png]
